# Clinical and preclinical evidence of sex-related differences in anthracycline-induced cardiotoxicity

**DOI:** 10.1186/s13293-018-0198-2

**Published:** 2018-08-29

**Authors:** Becky Meiners, Chetan Shenoy, Beshay N. Zordoky

**Affiliations:** 1 Department of Experimental and Clinical Pharmacology, College of Pharmacy, University of Minnesota, 308 Harvard St S.E, Minneapolis, MN 55455 USA; 2 Cardiovascular Division, Department of Medicine, University of Minnesota Medical School, Minneapolis, USA

**Keywords:** Anthracyclines, Doxorubicin, Cardiotoxicity, Sex, Male, Female

## Abstract

Anthracyclines are very effective chemotherapeutic agents that are widely used to treat pediatric and adult cancer patients. Unfortunately, the clinical utility of anthracyclines is limited by cardiotoxicity. There are several established risk factors for anthracycline-induced cardiotoxicity (AIC), including total cumulative dose, very young and very old age, concomitant use of other cardiotoxic agents, and concurrent mediastinal radiation. However, the role of sex as a risk factor for AIC is not well defined. In pediatric cancer patients, most studies support the notion that female sex is a significant risk factor for AIC. Conversely, there is anecdotal evidence that female sex protects against AIC in adult cancer patients. The lack of consistency in study designs and the different definitions of cardiotoxicity preclude reaching consensus regarding the role of sex as a risk factor for AIC in both pediatric and adult cancer patients. Therefore, more clinical research using reliable techniques such as cardiac magnetic resonance imaging is needed to determine if there truly are sex differences in AIC. In adult preclinical rodent studies, however, there is unequivocal evidence that female sex confers significant protection against AIC, with a possible protective effect of female sex hormones and/or a detrimental role of the male sex hormones. Although findings of these rodent studies may not perfectly mirror the clinical scenario in adult anthracycline-treated cancer patients, understanding the mechanisms of this significant sexual dimorphism may reveal important cardioprotective mechanisms that can be therapeutically targeted.

## Background

Anthracyclines (e.g., doxorubicin (DOX), Adriamycin®) are very effective chemotherapeutic agents that are widely used to treat both hematologic malignancies and solid tumors in pediatric and adult cancer patients [1, 2]. Unfortunately, the clinical utility of these highly effective agents is limited by cardiotoxicity that may lead to cardiac dysfunction and eventually heart failure [3]. Despite 40 years of research, the mechanism of anthracycline-induced cardiotoxicity (AIC) is still not fully understood. Nevertheless, a plethora of research has clearly demonstrated that there is no single mechanism that can fully explain all aspects of AIC. A significant body of evidence has suggested that oxidative stress plays a major role in AIC [4]. Anthracycline-induced mitochondriopathy and the resulting derailment in myocardial energetics have also been proposed to be major players in the pathogenesis of AIC [5]. More recently, topoisomerase II-beta has been determined to be a major molecular target of anthracyclines in the heart [6]. Interestingly, anthracyclines have been shown to induce all forms of cell death, namely, apoptosis, necrosis, and autophagy [7].

From a clinical perspective, AIC can be manifested in several forms. On one extreme is clinical heart failure with decreased left ventricular (LV) systolic function, and on the other extreme is an increase in serum biomarkers (troponin, natriuretic peptides) without symptoms or LV dysfunction [8]. In addition to cardiomyopathy, anthracyclines may also rarely cause arrhythmia [9], pericardial disease, and valvular abnormalities [10]. The precise incidence of various forms of AIC is not clear due to the various definitions used in research and clinical practice. There are several risk factors that have been found to increase the risk of AIC. First, both younger (pediatric) and older (geriatric) cancer patients are at increased risk of AIC. Pre-existing cardiovascular disease has been postulated to increase the risk of AIC in cancer patients. Co-administration of other cardiotoxic anti-cancer medication, e.g., trastuzumab (Herceptin®), and/or radiotherapy has also been shown to increase the risk of AIC [3]. However, the role of sex as a risk factor for AIC is controversial. In pediatric cancer patients, female sex has been traditionally considered a risk factor for AIC. However, the role of sex as a risk factor for AIC in adult cancer patients is inconclusive. On the other hand, preclinical animal research has demonstrated that adult female rodents are protected against AIC in a variety of rodent models.

In this review, we will discuss clinical and preclinical studies that reported sex-related differences in AIC. Understanding this sexual dimorphism is important from both the clinical and basic science perspectives. Clinically, it is very important for proper risk stratification of anthracycline-treated cancer patients. Although all anthracycline-treated patients should be monitored closely for AIC, risk stratification is important to guide the frequency and methodology of monitoring, to predict the maximum tolerated dose of anthracycline, to help decide whether to use a cardioprotectant drug, e.g., dexrazoxane or not, and to consider alternative chemotherapeutic agents in patients with very high risk for AIC [11]. From the basic science perspective, understanding the mechanisms of protection in female rodents may better elucidate the molecular mechanisms of AIC and identify protective pathways that can be targeted therapeutically.

## Clinical studies

### Clinical studies in pediatric cancer patients

Anthracyclines are clinically used to treat different types of malignancies in more than 50% of pediatric cancer patients, despite the known cardiotoxic effects of these medications [12]. With advances in the diagnosis and treatment of childhood cancer, the 5-year survival rate of most types of childhood cancer has dramatically increased over the past two decades, reaching more than 80% [13]. Although the increased survivorship is a cause for celebration, these young survivors are at increased risk of long-term adverse effects of their anti-cancer agents. The rate of heart failure is 15 times higher in childhood cancer survivors than in their siblings who did not have cancer [14]. AIC is definitely a major contributor to this increased risk of heart failure [13]. Indeed, pediatric cancer patients face potentially worse outcomes than adult patients as their hearts are still developing during the anthracycline exposure [15], since neonatal cardiomyocytes are more susceptible to DOX-induced apoptosis than terminally differentiated cardiomyocytes [16]. Similar to adult cancer patients, the total anthracycline cumulative dose is the major determinant of AIC. Therefore, every effort is made to limit the anthracycline dose to a minimum [17]. Nevertheless, recent research has shown that even very low anthracycline doses (≤ 100 mg/m^2^ DOX) can still cause subclinical cardiac damage, leaving those childhood cancer survivors with “weaker” hearts [18, 19].

A strong body of evidence suggests that girls are at higher risk for AIC than boys (Table 1). In a cohort of 150 patients who were treated with anthracyclines (65 girls and 85 boys, 7 to 29 years of age), Silber et al. first reported that girls are at increased risk of cardiac dysfunction than boys [20]. In this early study, the odds ratio (OR) for having an abnormal test result was 3.2 for females vs. males. The tests included resting and exercise gated nuclear angiography, using the standard electrocardiographic gating technique, and exercise testing using standard cycle geometry with ECG monitoring [20]. These findings were corroborated in a landmark study when Lipshultz et al. studied 120 children and adults who had been treated with DOX in childhood (58 males and 62 females). Significant sex-related differences were observed in this cohort. Increased LV dimensions and reduced LV mass were more predominant in female subjects, while reduced LV contractility, wall thickness, and fractional shortening were observed in both males and females [21]. The authors concluded that female sex is an independent risk factor for cardiac abnormalities after treatment with DOX in childhood cancer [21]. Although the mechanism of this sexual dimorphism was not identified, both studies proposed that differences in body composition between girls and boys may have contributed to the observed sexual dimorphism [20, 21]. They reasoned that girls have higher fat than boys leading to differences in DOX distribution in the body. Since DOX does not distribute to fat tissues, relatively higher DOX concentrations can be achieved in other tissues such as the heart [20, 21]. Of interest, sexual dimorphism in fat patterning was significant even in pre-pubertal 5–7-year-old children [22], which may contribute to the sex difference in AIC even in pre-pubertal pediatric cancer patients. In agreement with this notion, the clearance of doxorubicinol, the cardiotoxic metabolite of DOX, was lower in children with > 30% body fat [23]. However, in argument against this notion, DOX pharmacokinetic parameters were not different between boys and girls [24, 25]. In future studies, body composition, DOX, and doxorubicinol pharmacokinetics should be considered as confounding factors in multi-variate analysis to predict risk factors for AIC.

**Table 1:** Sex-related differences in anthracycline-induced cardiomyopathy in pediatric cancer patients

Pediatric studies	Number of patients	Age at diagnosis in years	Follow-up	Anthracycline cumulative dose	Major conclusion/comments
Silber et al. [20]	150	0.23–21, mean 9.5 years	0.09–18 years, average 4.7	50–750 mg/m^2^ Mean = 307 mg/m^2^	Female sex was a significant risk factor for cardiac abnormality (OR = 3.2)
Lipshultz et al. [21]	120	0.6–28.9	2–14.6	244–550 mg/m^2^ Median = 390 and 395 mg/m^2^ for osteogenic sarcoma and acute lymphoblastic leukemia, respectively	Female sex was an independent risk factor for cardiac abnormality after DOX therapy.
Krischer et al. [26]	6493	< 1 year to > 15 years	1 year	From < 99 mg/m^2^ to > 500 mg/m^2^ No specific doses were stated	Female sex increased the risk of anthracycline-associated cardiotoxicity (RR = 1.9) Review of protocols records
Ewer et al. [27]	113	1–17 Mean, 10.6; Median, 11	4–180 months	113–506 mg/m^2^ Mean = 341 mg/m^2^	More girls than boys developed cardiac dysfunction (not statistically significant, small sample size)
Green et al. [28]	2710	< 16	Up to 20 years	59–691 mg/m^2^	Females are at 4 times higher risk of congestive heart failure after Review of medical records
Pein et al. [34]	229	0–21	> 15 years	40–600 mg/m^2^ Mean = 344 mg/m^2^	Sex was not a significant risk factor
Van Dalen et al. [35]	830	0.1–18.0 years, median 8.7 years	0.01–28.4 years, median 7.1 years	15–900 mg/m^2^ Mean = 288 mg/m^2^ Median = 280 mg/m^2^	Sex was not a significant risk factor for clinical heart failure (female RR = 1.46, 95% CI 0.62–3.43 *p* value = 0.39) Review of medical records/registry charts
Hudson et al. [36]	217	5 years	3–21.7 years	25–510 mg/m^2^ Median = 202 mg/m^2^	Sex was not a significant risk factor for abnormal non-invasive cardiac testing
Armstrong et al. [29]	20,483	0–20 years	> 5 years	NA	Female sex was a risk factor for mortality due to cardiac disease (RR = 1.4). Reviewing of national death index and death certificates
Mulrooney et al. [10]	14,358	0–20	> 5 years	NA	Female sex was a risk factor for congestive heart failure (HR = 1.4) Retrospective cohort study
Lipshultz et al. [30]	66 (out of 100 enrolled patients)	Mean age 7.8 years	5 years	300 mg/m^2^	Reduction in left ventricular fractional shortening was significantly greater in DOX-treated girls than boys
Rathe et al. [37]	80	0.8–13.4 Median of 4 years	1.1– 20.6 Median of 8.2 years	120–300 mg/m^2^	Sex was not a significant risk factor for subclinical decline in cardiac function (less than 300 mg/m^2^ DOX) Longitudinal follow-up
Amigoni et al. [31]	62	0–13	6–19 years	120–280 mg/m^2^ Mean = 228 mg/m^2^	Left ventricular mass and dimensions were reduced only in female ALL survivors (less than 300 mg/m^2^ DOX)
Andolina et al. [38]	308	< 1 year to > 5 years	≥ 5 years	Mean = 210 mg/m^2^ Median = 190 mg/m^2^	Sex was not a significant risk factor Retrospective medical record and echocardiography review
Van der Pal et al. [39]	525	9	≥ 5 years	33–720 mg/m^2^ Median = 250 mg/m^2^	Sex was not a significant risk factor
Brouwer et al. [40]	277	≤ 20 years Median 8.8	5–31 years Median 18.2	50–600 mg/m^2^ Median = 183 mg/m^2^	Female sex was protective from diastolic dysfunction (OR = 0.3). Sex was not a risk factor for systolic dysfunction
Vandecruys et al. [18]	37	0.2–12.2 Median 4.8	10.6–18.3 Median 13.3	180–240 mg/m^2^	Subclinical echocardiographic abnormalities were found more frequently in male ALL survivors who had received less than 250 mg/m^2^ DOX
Toro-Salazar et al. [32]	46	Mean of 11 ± 5.1	2.5–26.9	200–600 mg/m^2^ Mean = 328 mg/m^2^	Higher extracellular volume was detected in female than in male survivors who had received 200 mg/m^2^ or more
Ylanen et al. [41]	62	0–13.8	4.9–18	80–419 mg/m^2^ Median = 222 mg/m^2^	There was a trend toward a male predominance among those with abnormal left ventricular ejection fraction and those with abnormal right ventricular ejection fraction
Tham et al. [33]	30	7–19 years	7.6 ± 4.5	80–375 mg/m^2^ Mean = 197.2 mg/m^2^	Higher non-contrast myocardial T1 and extra-cellular volume (ECV) was observed in female subjects

In another study, cardiotoxic events were determined by a review of protocol records in 6493 children with cancer who had received anthracycline chemotherapy on Pediatric Oncology Group (POG) protocols from 1974 to 1990. Although early clinical cardiotoxicity was rare in children treated with anthracyclines, female sex was found to increase the relative risk of AIC (RR = 1.9) [26]. Similarly, more girls than boys developed cardiac dysfunction and more girls died of progressive cardiac failure after 4–180 months of receiving DOX; however, the difference was not statistically significant [27]. Medical charts review of 2710 pediatric cancer patients who had received DOX revealed that females were at a four times higher risk for congestive heart failure than boys [28]. In a study that assessed late mortality in childhood cancer survivors, female sex was found to be a risk factor for mortality due to cardiac disease [29]. Similarly, a retrospective cohort study showed that female sex was a risk factor for congestive heart failure in childhood cancer survivors (RR = 1.4) [10]. Intriguingly, female sex was protective against myocardial infarction (RR = 0.6), which was not associated with the use of anthracyclines [10]. In a randomized clinical trial to assess the protective effect of dexrazoxane against AIC, the reduction in LV fractional shortening was significantly greater in girls than in boys in the DOX-only group [30].

Since the recognition of the dose-dependent cardiotoxicity of anthracyclines, more recent clinical protocols adopted a lower cumulative anthracycline dose (usually less than 300 mg/m^2^). Therefore, recent studies have investigated sex-related differences in AIC within this dose range. Amigoni et al. studied sex differences in AIC in acute lymphocytic leukemia survivors who had received smaller cumulative doses of DOX (< 300 mg/m^2^) [31]. In a small cohort of 62 survivors, LV mass and dimensions were reduced only in females [31]. In asymptomatic childhood cancer survivors who had been treated with anthracycline, two studies have reported higher mean extra-cellular volume in female survivors than in males, suggesting higher diffuse interstitial myocardial fibrosis and higher collagen volume fraction in female subjects [32, 33]. Although these parameters represent early tissue markers of ventricular remodeling [33], it is still not known whether they can predict clinically significant cardiac dysfunction on the long term.

In contrast to the aforementioned studies, there are a number of studies that did not find female sex as a risk factor for AIC. In a cohort of 229 childhood cancer survivors with a minimum follow-up of 15 years, Pein et al. did not find female sex to be an independent risk factor for cardiac failure or asymptomatic cardiac abnormalities [34]. Similarly, female sex was not identified as a risk factor for anthracycline-induced clinical heart failure after reviewing the medical records or registry charts of 830 survivors [35]. In this study, the cumulative anthracycline dose was the only significant risk factor for anthracycline-induced clinical heart failure [35]. In a cohort of asymptomatic long-term childhood cancer survivors, female sex was not identified as a risk factor for abnormal non-invasive cardiac testing that included measuring fractional shortening and after-load [36]. Similarly, cumulative anthracycline dose was the only significant risk factor for anthracycline-induced reduction in fractional shortening [36]. In a longitudinal follow-up study, female sex was not identified as a risk factor for the deterioration of ejection fraction in childhood cancer survivors who had received anthracycline therapy of 300 mg/m^2^ or less [37]. Nevertheless, LV mass was significantly decreased in female subjects only, while male sex was significantly associated with diastolic dilatation of left ventricle and was a significant risk factor for reduced ejection fraction by univariate analysis [37]. In a retrospective record review, female sex was not identified as a risk factor for abnormal echocardiogram [38]. The cumulative anthracycline dose was the only significant risk factor for an abnormal echocardiogram in this study [38]. In another study, female sex was not a risk factor for cardiac dysfunction; nevertheless, other risk factors such as higher cumulative anthracycline dose, younger age at diagnosis, and chest radiation were identified [39].

On the other end of the spectrum, a few studies have reported that female sex may be protective against AIC in pediatric cancer patients. For instance, female sex was a potential confounder for diastolic dysfunction with OR of 0.3 (95% CI 0.1–097), suggesting a probably protective effect of female sex; however, systolic function was not different between male and female childhood cancer survivors [40]. In a small number of acute lymphocytic leukemia survivors, systolic (fractional shortening and ejection fraction) and diastolic function (A maximum and E/A ratio) were declined in male survivors as compared to their respective controls. These differences could not be detected in females [18]. Nevertheless, the figures for these sex differences did not reach statistical significance, and the authors of this study attributed this observation to the small number of participants in their study [18]. In another small study, there was a trend (*p* = 0.053) toward a male predominance among those with abnormal LV ejection fraction (defined as LVEF < 45%). Similarly, more male subjects than females had abnormal right ventricular ejection fraction (*p* = 0.057) [41]. Similar to Vandecruys et al., these findings may be attributed to the small number of study participants and may not represent a true higher risk for AIC in male childhood cancer survivors. In a recent meta-analysis of data on clinical heart failure in 14 osteosarcoma trials, the authors found that female sex had significant protective effect on cardiotoxicity [42].

### Clinical studies in adult cancer patients

In contrast to the many studies on sexual dimorphism of AIC in pediatric cancer patients, there is much less research documenting sex differences in adult cancer population (Table 2). We think this is because the majority of research done in the area of AIC in adults is in breast cancer patients, primarily a female-only population. In a few studies of hematological malignancies, both sexes were treated with anthracyclines allowing the study of sex differences. In patients with Hodgkin lymphoma, the risk of hospital admission for cardiac disease was evaluated in 615 patients treated with DOX, mediastinal radiation, or a combination thereof [43]. Male sex was identified as a significant risk factor for adverse cardiac events [43]. The authors attributed this to a higher baseline cardiac disease in males. Intriguingly, DOX alone did not increase the risk of adverse cardiac events in this study; however, patients treated with DOX and mediastinal radiation had a significantly higher risk of cardiac hospitalization compared with the general population and those receiving mediastinal radiation without DOX [43]. Similarly, male sex was a significant risk factor for cardiac hospitalization among Hodgkin lymphoma patients in another study [44].

**Table 2:** Sex-related differences in anthracycline-induced cardiomyopathy in adult cancer patients

Adult studies	Number of patients	Age at diagnosis	Time after chemo-therapy	Anthracycline dose	Major conclusion/comments
Von Hoff et al. [82]	4018	Mainly adult population (10% < 18 years)	0–231 days	13–5047 mg/m^2^ Mean = 240 mg/m^2^ Median = 183 mg/m^2^	No effect of sex
Hrushesky et al. [83]	34	19–78	2 years	300–550 mg/m^2^	Higher ratio of females developed CHF (7 out of 24, compared to 1 out of 10) Confounding factors of sex-dependent diagnosis and DOX dose.
Clements et al. [45]	33	Mean age of 55 ± 14	Within 1 year after the onset of treatment	40–618 mg/m^2^ Mean = 223 mg/m^2^	Male sex was an independent risk factor for development of systolic and diastolic cardiac dysfunction early after initiation of DOX
Limat et al. [84]	151	25–79 Median age of 59	1 year	50–400 mg/m^2^ Median = 290 mg/m^2^	No effect of sex
Hequet et al. [46]	141	15–69 Median 47	At least 5 years	250–550 mg/m^2^ Median = 300 mg/m^2^	Male sex was a risk factor for subclinical cardiomyopathy
Elbl et al. [85]	47	18–76 Mean of 49	1 year	50–400 mg/m^2^ Mean = 277 mg/m^2^	Sex was not a risk factor for either drop in EF > 10% or all cardiac events
Hershman et al. [50]	4001	> 65	8 years	NA	No difference (elderly population)
Neilan et al. [49]	91	Mean of 43	A median of 88 months	Mean = 276 mg/m^2^	No significant effect of gender on major adverse cardiac events
Szmit et al. [47]	208	Median of 56	After the last administered cycle of chemotherapy	300–400 mg/m^2^ Median = 350 mg/m^2^	Female sex was protective for left ventricular systolic dysfunction (OR = 0.324)
Wang et al. [48]	2285	Mean of 53	193–1666 days	Doxorubicin 88–267 mg/m^2^ Epirubicin 100–295 mg/m^2^ Idarubicin 8–19 mg/m^2^	Male gender hazard ratio of 1.84 for cardiac events (symptomatic heart failure or cardiac death)

In a small number of patients, Clements et al. evaluated LV systolic and diastolic function before and after DOX therapy to assess the early impact on the heart [45]. The decrease in LVEF was significantly greater in men than in women [45]. Male sex was identified as a significant independent predictor for the decline in LVEF after DOX therapy [45]. Similarly, male sex was identified as a risk factor for subclinical late cardiomyopathy in adult lymphoma patients [46]. Other risk factors were older age, higher cumulative DOX dose, radiotherapy, and being overweight [46].

In a study that evaluated the risk factors for the development of early LV systolic dysfunction directly after anthracycline-based chemotherapeutic regimen in adult patients with lymphoma, female sex was protective against LV systolic dysfunction (OR = 0.324; 95% CI 0.106–0.989) [47]. Similarly, in a large cohort of patients receiving anthracyclines for different types of malignancies, major adverse cardiac events (symptomatic heart failure or cardiac death) were more frequent in men than in women (*p* = 0.03) [48].

On the other hand, in a small cohort of patients with anthracycline-induced cardiomyopathy, reduced LV mass has been observed in both men and women [49]. In elderly patients (≥ 65 years) diagnosed with diffuse large B-cell lymphoma and received DOX chemotherapy, sex was not a risk factor for subsequent congestive heart failure [50]. It is important to mention that the risk of cardiovascular diseases is similar in post-menopausal women to age-matched men [51]; therefore, it is likely that similar baseline cardiovascular health in elderly men and women leads to similar susceptibility to AIC.

## Preclinical studies

### Juvenile animal models

There is rarity of preclinical animal models of juvenile DOX-induced cardiotoxicity, as compared to adult animal studies [15, 52–57]. In these few studies, sex was not considered as a biological variable in either the design or the analysis of their findings. To the best of our knowledge, there is one study that evaluated the toxicological profile of DOX in juvenile mice, wherein DOX administration (3 mg/kg/day intra-peritoneal on post-natal days 13, 17, 20, 35, 39, and 42) caused significant mortality in both male and female mice. DOX caused a significant reduction in heart weight in both male and female mice, without significant histopathological changes, due to early sacrifice of DOX-treated mice [58]. The study was interrupted at post-natal day 73 due to the moribund conditions of DOX-treated animals, precluding the evaluation of possible late onset cardiotoxicity [58]. There are a number of limitations in the study of sex differences of AIC in juvenile animal models. First, clinically relevant animal models of AIC should use smaller repeated doses of DOX over a prolonged period of time [59]. Signs of sexual maturity have been detected in rodents as early as 4 weeks of age [60]; therefore, the prolonged duration of chronic DOX administration will be likely to extend beyond the sexual maturity of the animals. Second, intra-venous administration of anthracycline is also recommended for preclinical animal models of AIC to mirror the clinical scenario [59]. This is also technically challenging in juvenile mice especially before weaning. Taken together, rodents may not be the best animal model to study sex differences of juvenile AIC, and other animal models, e.g., miniature pigs, may better address this research question.

### Adult animal models

Sex-related differences have been well documented in adult animal models of DOX-induced cardiotoxicity. In all studies of acute and chronic models of AIC, female rodents were markedly protected against cardiotoxicity, as compared to males (Table 3). A number of mechanisms have been postulated to explain this sexual dimorphism.

**Table 3:** Sex-related differences in anthracycline-induced cardiomyopathy in preclinical adult animal models

Study	Species	DOX dose	Major findings/proposed mechanisms
Julicher et al. [76]	Adult Lou/M Wsl rats	Chronic 1 mg/kg for 5 consecutive days, then weekly	Female rats were protected from DOX-induced cardiotoxicity, nephrotoxicity, and hepatotoxicity.
van Almen et al. [86]	Adult WT and thrombospondin-2 KO mice	Chronic 2 mg/kg/week for 12 weeks	Female sex protected against DOX-induced myocardial fibrosis in the KO mice.
Zhang et al. [66]	Adult SHR	Chronic 1 mg/kg/week for 9, 10, or 12 weeks	Female sex is protected against DOX-induced cardiotoxicity and nephrotoxicity. Ovariectomy prevented this protection. Mechanism: cardiac mast cells activity
Moulin et al. [61]	Adult Wistar rats	Chronic 2 mg/kg/week for 7 weeks	Female sex protected against DOX-induced cardiotoxicity. No mortality in females, up to 50% mortality in males. Mechanism: mitochondrial dysfunction and AMPK signaling
Moulin et al. [62]	Adult Wistar rats	Chronic 2 mg/kg/week for 7 weeks	Sexual dimorphism of cardiac phospholipid content
Gonzalez et al. [64]	Adult SST-2 tumor-bearing SHR	Acute 10 mg/kg once	Adult tumor-bearing male SHRs are more cardiosensitive to Dox than female or hormone-deficient animals
Zordoky et al. [67]	Adult WKY and SHHF rats	Chronic 2 mg/kg/week for 8 weeks	Female sex protected against DOX-induced hypertension in WKY rats and protected against DOX-induced cardiac dysfunction in SHHF rats. Female sex protected against DOX-induced nephrotoxicity in both strains.
Jenkins et al. [65]	Young adult B6C3F1 mice (8 weeks)	Chronic 3 mg/kg/week for 6, 7, 8, and 9 weeks	Female sex protected against DOX-induced myocardial injury
Grant et al. [68]	Adult C57Bl/6 mice	Acute 20 mg/kg once	Adult male mice are more susceptible to DOX-induced cardiotoxicity. Possible involvement of CYP450 differential expression

In a chronic model of DOX-induced cardiotoxicity in adult Wistar rats, DOX administration caused 50% mortality in male rats, while female rats survived and had normal appearance after 7 weeks of weekly IV injections of 2 mg/kg DOX [61]. Several features of DOX-induced cardiomyopathy were more pronounced in male than in female rats, including cardiac atrophy, reduced LVEF, myocardial fibrosis, myolysis, and upregulated gene expression of atrial natriuretic factor and inflammatory markers [61]. Mechanistically, DOX severely impaired mitochondrial biogenesis and downregulated the energy-sensing AMP-activated protein kinase in hearts of male, but not female, rats implying that DOX-induced mitochondrial dysfunction and altered myocardial bioenergetics may have contributed to sexual dimorphism of chronic DOX-induced cardiotoxicity [61]. DOX administration has also induced male-specific cardiac cardiolipin remodeling with a decreased expression of genes involved in the biosynthesis of fatty acids [62]. Interestingly, there was no difference in death signaling and oxidative stress between male and female animals, suggesting that these pathways are not primarily involved in the observed sexual dimorphism [61]. In agreement with this finding, there were no major sex differences in apoptosis signaling after administration of a single DOX dose (10 mg/kg) to Fisher 344 rats [63]. Similarly, gene expression analysis in hearts of DOX-treated tumor-bearing spontaneously hypertensive rats (SHRs) revealed significant differential expression of mitochondria-related oxidative stress genes in male hearts, but not in females, with no difference in apoptosis-related signaling pathways [64].

In B6C3F_1_ mice, chronic DOX administration (3 mg/kg/week IV for 6, 7, 8, and 9 weeks) revealed a greater susceptibility of male mice to DOX-induced cardiotoxicity compared to females as manifested by lower heart weights, higher plasma cardiac troponin T concentrations, and more severe cytoplasmic vacuolization [65]. In contrast to the finding that DOX induces equal apoptosis in hearts of both male and female rodents [61, 63, 64], higher number of TUNEL-positive cardiomyocytes was observed in DOX-treated male mice compared to DOX-treated female mice, suggesting that apoptosis plays a role in defining sexual dimorphism of DOX-induced cardiotoxicity [65].

In another study, chronic DOX administration (1 mg/kg/week for 9 weeks) to adult male SHRs caused more severe cardiomyopathy than in females [66]. The increased cardiotoxicity was associated with higher number of cardiac mast cells and percentage of cardiac mast cells undergoing degranulation, suggesting that cardiac mast cell activity could have contributed to the observed sexual dimorphism [66]. In contrast, there was no significant difference in the number or morphology of cardiac mast cells between DOX-treated male and female B6C3F_1_ mice [65].

Pre-existing cardiovascular disease is a significant risk factor for more severe AIC [3]; therefore, we studied the interplay between genetic predisposition to cardiovascular diseases and sexual dimorphism of AIC [67]. We demonstrated that female sex was protective against DOX-induced increase in systolic blood pressure in control WKY rats, while it protected against the decline in cardiac function and the increase in serum troponin level in spontaneously hypertensive heart failure-prone rats, suggesting an interaction between sexual dimorphism and genetic predisposition to cardiovascular diseases [67].

We have also shown that acute DOX-induced cardiotoxicity was also more marked in adult male C57Bl/6 mice than in females [68]. This was accompanied by sex-specific alteration of a number of cytochrome P450 genes involved in arachidonic acid metabolism [68]. Since perturbations in cytochrome P450-mediated arachidonic acid metabolism have been implicated in the pathogenesis of several cardiovascular diseases including AIC [69–71], sex-dependent alteration in these enzymes may also contribute to the observed sexual dimorphism of acute DOX-induced cardiotoxicity. Of importance, we demonstrated that acute DOX cardiotoxicity caused a significant induction of the apoptotic marker, BAX/Bcl-2 gene expression in hearts of both male and female mice 24 h after DOX, but in hearts of only male mice 6 days after DOX, implying that sexual dimorphism of DOX-induced apoptosis may be time-dependent. This observation may explain the discrepancy between some studies that reported equal apoptosis in hearts of male and female DOX-treated animals and those studies that reported a sexually dimorphic apoptosis.

To identify the role of sex hormones in driving sexual dimorphism of DOX cardiotoxicity, a number of studies investigated the cardiotoxic effects of DOX in ovariectomized female and castrated male animals. In ovariectomized female SHRs, the severity of chronic DOX-induced cardiotoxicity was similar to that in male animals and markedly higher than that in intact females [66]. DOX-induced cardiotoxicity in ovariectomized SHRs was accompanied by a significantly higher number of cardiac mast cells in ovariectomized DOX-treated SHRs compared to DOX-treated intact females [66]. Similarly, ovariectomy has been shown to exacerbate acute DOX-induced cardiotoxicity in female Wistar rats, which was associated with an exacerbation of DOX-induced oxidative stress [72]. Importantly, administration of 17-β-estradiol or the catechoestrogens (2- and 4-hydroxyestradiol) reduced all parameters of cardiac injury and oxidative stress in DOX-treated ovariectomized rats [73, 74]. Similarly, a recent study has shown that exogenous 17-β-estradiol administration suppressed DOX-induced cardiotoxicity in ovariectomized female tumor-bearing SHR rats. Surprisingly, co-administration of progesterone negated the protective effect of 17-β-estradiol [75]. In contrast to these findings, Gonzalez et al. showed that ovariectomy did not exacerbate acute DOX-induced cardiotoxicity in tumor-bearing SHRs [64]. Intriguingly, they demonstrated that castration protected male tumor-bearing SHRs from acute DOX-induced cardiotoxicity [64], implying a detrimental role of male sex hormones rather than a protective role of female sex hormones.

In addition to sexual dimorphism of DOX-induced cardiotoxicity, a number of studies have also reported marked sex differences in DOX-induced nephrotoxicity in rodent animal models, wherein female rodents are protected against DOX-induced nephrotoxicity, as compared to males [67, 76]. Although anthracyclines have been reported to cause nephrotoxicity in cancer patients [77, 78], anthracycline-induced nephrotoxicity is more severe in rodents [67]. Intriguingly, we demonstrated that the extent of sexual dimorphism was more prominent in DOX-induced pathological lesions in the kidney than those in the heart in both WKY and SHHF rats [67]. Since the cardiovascular-renal axis is an important factor in cardiovascular health and disease [79], sexual dimorphism of DOX-induced nephrotoxicity may have been overlooked in some rodent studies of DOX cardiotoxicity.

Similar to the reviewed clinical studies, the aforementioned preclinical studies used different doses, dosage schedules, and routes of administration of DOX in different species/strains of experimental animals. While most of the studies were conducted in non-tumor-bearing animals, some were conducted in tumor-bearing animals. Although this marked heterogeneity of study designs may preclude reaching consensus regarding the exact sex-related phenotypic differences and molecular mechanisms of AIC, there is strong evidence that adult female rodents are significantly protected against AIC. Indeed, the degree of the sexual dimorphism in these animal studies does not mirror the subtle sex-related differences reported in clinical studies. In most rodent strains, males have much higher fat than age-matched females [80]. This sexual dimorphism in body composition may exacerbate AIC in male rodents. Intriguingly, sexual dimorphism in body composition in rodents is opposite to what is reported in humans, i.e., male rodents have higher fat, while male humans have lower fat than females [80, 81]. This may explain the discrepancy between animal and human data with regard to AIC. Future studies should be conducted in rodent strains which show similar body composition phenotype to humans, e.g., the KK/H1J mouse strain.

## Conclusion

Although a plethora of clinical studies attempted to determine the role of sex as a risk factor for AIC, the clinical data are still inconclusive. There is anecdotal evidence that female sex is a risk factor for AIC in pediatric cancer patients, while it may be protective in adult cancer patients. The lack of consistency in study designs and the different definitions of cardiotoxicity preclude reaching consensus regarding the role of sex as a risk factor in AIC. In addition, the divergence in findings may be explained by the limitations of diagnosing AIC, especially in children. Therefore, more research using reliable techniques, such as cardiac magnetic resonance imaging, is needed to determine if there truly are sex differences in AIC. In addition, prospective controlled studies will provide stronger evidence for risk factors for AIC. With regard to preclinical studies, there is lack of studies that investigate sex difference in juvenile experimental animals. Conversely, there is strong evidence that female sex markedly protects against AIC in adult rodents. Although findings of these rodent studies may not mirror the clinical scenario in adult anthracycline-treated cancer patients, understanding the mechanisms of this significant sexual dimorphism may reveal important cardioprotective mechanisms that could be therapeutically targeted.

## Abbreviations

AIC: Anthracycline-induced cardiotoxicity
DOX: Doxorubicin
EF: Ejection fraction
LV: Left ventricle
SHR: Spontaneously hypertensive rat
